# Influence of Segmentation of Ring-Shaped NdFeB Magnets with Parallel Magnetization on Cylindrical Actuators

**DOI:** 10.3390/s140713070

**Published:** 2014-07-21

**Authors:** Paulo Roberto Eckert, Evandro Claiton Goltz, Aly Ferreira Flores Filho

**Affiliations:** 1 Post-Graduate Program in Electrical Engineering, Federal University of Rio Grande do Sul, Av. Osvaldo Aranha 103, CEP: 90035-190, Porto Alegre, RS, Brazil; E-Mail: aly.flores@ufrgs.br; 2 Technology Center, Federal University of Santa Maria, Av. Roraima 1000, CEP: 97105-900, Santa Maria, RS, Brazil; E-Mail: evandro@inf.ufsm.br

**Keywords:** permanent magnets, magnetization pattern, parallel magnetization, radial magnetization, NdFeB magnets, linear cylindrical electric machine, segmented ring-shaped magnets

## Abstract

This work analyses the effects of segmentation followed by parallel magnetization of ring-shaped NdFeB permanent magnets used in slotless cylindrical linear actuators. The main purpose of the work is to evaluate the effects of that segmentation on the performance of the actuator and to present a general overview of the influence of parallel magnetization by varying the number of segments and comparing the results with ideal radially magnetized rings. The analysis is first performed by modelling mathematically the radial and circumferential components of magnetization for both radial and parallel magnetizations, followed by an analysis carried out by means of the 3D finite element method. Results obtained from the models are validated by measuring radial and tangential components of magnetic flux distribution in the air gap on a prototype which employs magnet rings with eight segments each with parallel magnetization. The axial force produced by the actuator was also measured and compared with the results obtained from numerical models. Although this analysis focused on a specific topology of cylindrical actuator, the observed effects on the topology could be extended to others in which surface-mounted permanent magnets are employed, including rotating electrical machines.

## 1. Introduction

Nowadays there is a growing concern about power efficiency and high force density actuators to produce linear movement, for example in industrial applications and active suspension [1,2]. A class of actuators that fits these needs are the cylindrical actuators with built in NdFeB permanent magnets (PMs). The simple concept makes it easy to design these actuators with high power efficiency and with high force density [3]. Generally, they employ ring-shaped magnets, ring-shaped coils and ferromagnetic cores.

**Figure 1.**
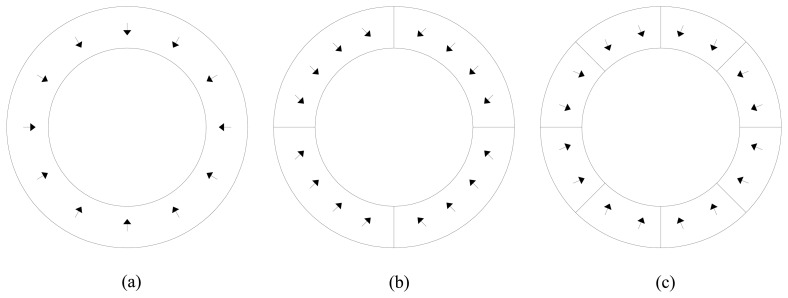
Permanent magnets with (**a**) ideal radial magnetization; (**b**) parallel magnetization with four segments forming a ring; and (**c**) parallel magnetization with eight segments forming a ring.

The ring-shaped NdFeB permanent magnets are in practice however very difficult to magnetize in an ideal radial pattern, as shown in Figure 1a. Moreover, for each different permanent magnet shape and size a specific magnetizing fixture must be employed. Alternatively, ring segmentation and parallel magnetization, such as in the examples shown in Figure 1b,c, are often preferred since it reduces costs and results in an easy way of manufacturing the permanent magnets [4,5].

The use of segmentation and parallel magnetization is mentioned in some papers [6–8]. However, the number of segments can have a significant influence on the performance of a linear cylindrical slotless actuator, and for this reason its effects should be evaluated. Although some fixtures to radially magnetize NdFeB PMs were proposed in the literature [9,10], in [4] it was shown that an ideal radial magnetization is in practice difficult to achieve, if ever possible. It was mentioned that segmentation and parallel magnetization is an option; however, no evaluation on the performance of the actuator as a function of the number of segments was presented. In [5,11] the influence that the number of segments with parallel magnetization has on actuators with quasi-Halbach arrays was discussed, although no experimental results were presented.

In order to bring together the analysis of the effects that segmentation followed by parallel magnetization has on cylindrical slotless actuators with experimental validation, this work studies the effects of the number of segments with parallel magnetization on the distribution of magnetic flux density and on axial force using analytical modeling of magnetization, 3D Finite Element Analysis (FEA) and measurements on a prototype.

In Section 2, the topology under study as well as geometrical description and characteristics of the materials employed to build the prototype of the actuator are presented. In Section 3, the mathematical model of magnetization for parallel and radial magnetization for this topology is demonstrated. This includes an evaluation of the effects that the number of segments has on the mean value of the radial component, on the peak value and on the root mean square (RMS) value of the circumferential component of magnetization. In Section 4, the 3D FEA model using symmetry, is presented. In addition, results of radial and tangential components of magnetic flux density and results of force as a function of the number of segments are presented. Also, the influence that the armature reaction, the pole pitch in axial direction and the length of the air gap have on such actuators with parallel magnetization is shown. Finally, in Section 5, experimental setups for measurements, results for flux density in the air gap and results of linear static force are presented.

## 2. Prototype

**Figure 2.**
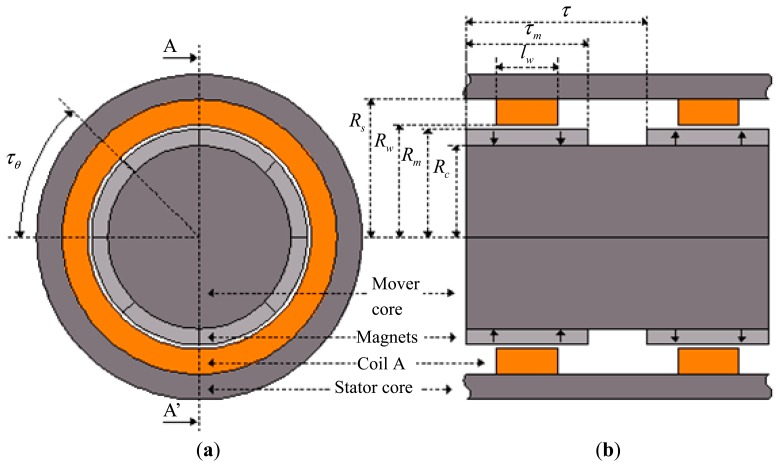
Linear cylindrical actuator showing (**a**) axial view and (**b**) view of section AA′.

The prototype is a cylindrical linear actuator that employs NdFeB permanent magnets as shown in Figure 2. The actuator has two poles, each one built with eight segments with parallel magnetization, forming a ring. The stator core and the mover core are made of soft ferromagnetic material, *i.e.*, AISI 1020 steel.

Two ring-shaped coils, each one with 64 turns, are made of standard 12 AWG enameled copper wire. Each coil is positioned in front of the corresponding pole face of the permanent magnet, producing force in the axial direction when electric current is properly applied to them. These coils are connected in series in a way that the current is established in the coils in opposite directions with respect to each other. The actuator has only one phase, making it simple to control its position and force. However, this configuration could easily be extended to a three-phase configuration leading to a higher force density [3]. The geometrical parameters shown in Figure 2 are listed in Table 1.

**Figure 3.**
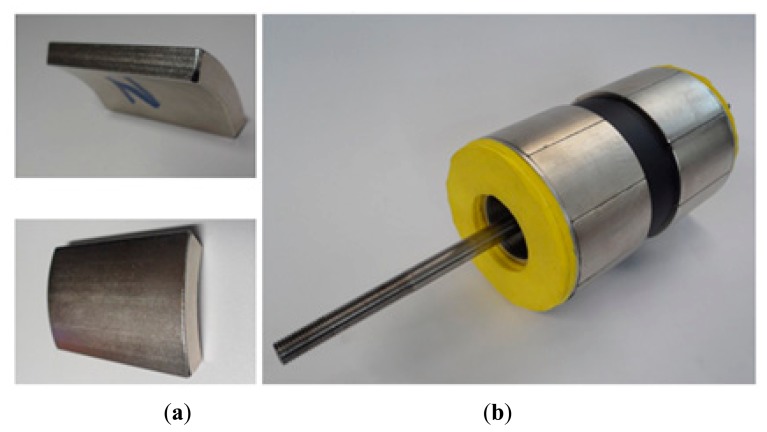
Picture of (**a**) a 45° segment of magnet with parallel magnetization employed to build the prototype and (**b**) the mover of the prototype built with two poles of eight segments of permanent magnets with parallel magnetization.

The permanent magnets of the prototype have 45° arcs as shown in the pictures of Figure 3a. The moving part of the prototype, with two magnetic poles and with the permanent magnets already attached to the core, can be seen in Figure 3b. The yellow parts are bumpers employed to attenuate any eventual mechanical impacts.

Geometrical details and magnetic characteristics of the permanent magnets are listed in Table 2. Based on the geometrical and magnetic characteristics of that actuator, an analytical model of magnetization, an analysis of the magnetic flux density, and an analysis of the static axial force produced by the actuator were evaluated as described in the following sections.

## 3. Analytical Description of Magnetization

Ideal radial magnetization would result in a component in the radial direction only. Therefore, an equation to describe this component of magnetization along the axial axis, *i.e.*, *M_r_*(*z*), can be obtained [11]. For the topology shown in Figure 2 and its parameters it results in:

(1) $$M_{r}\left(z\right)=\frac{B_{\text{rem}}}{\mu_{0}}\sum_{\text{n}=1}^{\infty }\frac{4}{n\pi }\sin\left(\frac{\tau_{m}n\pi }{2\tau }\right)\sin\left(\frac{n\pi }{2}\right)\sin\left(\frac{n\pi z}{\tau }\right)$$

where *B_rem_* and *μ*_0_ are the remanent flux density of the permanent magnets and the permeability of the vacuum respectively. When the actuator is formed by segmented rings with parallel magnetization, it presents radial, *M_r_*(*θ*,*z*), and circumferential, *M_θ_*(*θ*,*z*), components [11], which leads to a distribution that can be described by:

(2) $$M_{r}(\theta ,z)=\frac{B_{\text{rem}}}{\mu_{0}}\sum_{\text{m}=0}^{\infty }M_{rm}\cos\left(\frac{m\pi \theta }{\tau_{\theta }}\right)\sum_{\text{n}=1}^{\infty }\frac{4}{n\pi }\sin\left(\frac{\tau_{m}n\pi }{2\tau }\right)\sin\left(\frac{n\pi }{2}\right)\sin\left(\frac{n\pi z}{\tau }\right)$$

(3) $$M_{\theta }\left(\theta ,z\right)=\frac{B_{\text{rem}}}{\mu_{0}}\sum_{\text{m}=0}^{\infty }M_{\theta m}\sin\left(\frac{m\pi \theta }{\tau_{\theta }}\right)\sum_{\text{n}=1}^{\infty }\frac{4}{n\pi }\sin\left(\frac{\tau_{m}n\pi }{2\tau }\right)\sin\left(\frac{n\pi }{2}\right)\sin\left(\frac{n\pi z}{\tau }\right)$$

where coefficients *M_rm_* and *M_θm_* are given by:

(4) $$M_{rm}=\{\begin{array}{ll} \frac{NS\sin\left(\overset{\pi }{\;}/\underset{NS}{\;}\right)}{\pi } & \text{for}\;m=0\\ 0 & \text{for}\;m=\overset{2}{\;}/\underset{NS}{\;}\\ \frac{-8\pi \cos{\left(\overset{m\pi }{\;}/\underset{2}{\;}\right)}^{2}{\left(-1\right)}^{n}\sin\left(\overset{\pi }{\;}/\underset{NS}{\;}\right)}{NS\left({\left(n\pi \right)}^{2}-{\left(\overset{2\pi }{\;}/\underset{NS}{\;}\right)}^{2}\right)} & \text{for}\;m\neq \overset{2}{\;}/\underset{NS}{\;} \end{array}$$

(5) $$M_{\theta m}=\{\begin{array}{ll} 0 & \text{for}\;m=0\\ 0 & \text{for}\;m=\overset{2}{\;}/\underset{NS}{\;}\\ \frac{4n\pi \cos{\left(\overset{m\pi }{\;}/\underset{2}{\;}\right)}^{2}\sin\left(\overset{\pi }{\;}/\underset{NS}{\;}\right)}{{\left(n\pi \right)}^{2}-{\left(\overset{2\pi }{\;}/\underset{NS}{\;}\right)}^{2}} & \text{for}\;m\neq \overset{2}{\;}/\underset{NS}{\;} \end{array}$$

where *NS* is the number of segments.

**Figure 4.**
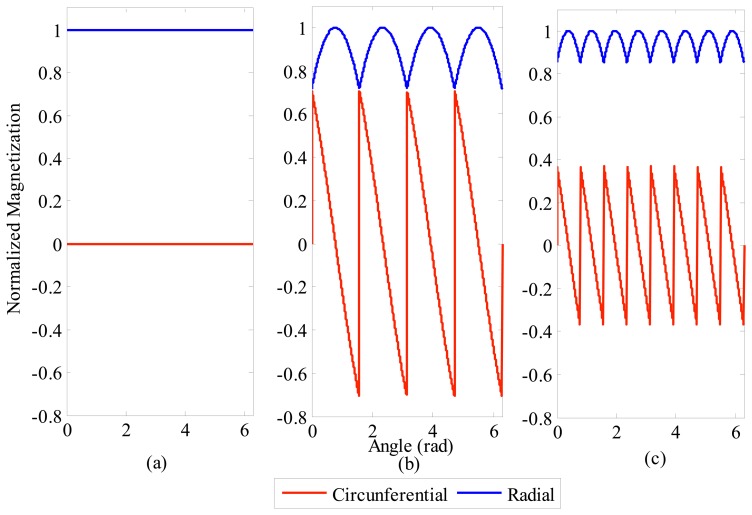
Normalized radial and circumferential magnetization components of ring-shaped magnets with (**a**) ideal radial magnetization, (**b**) four segments with parallel magnetization, and (**c**) eight segments with parallel magnetization.

From Equations (1)–(3), the radial and circumferential components of magnetization for any number of segments can be predicted. Figure 4 shows the results for ideal magnetization, for four segments and for eight segments, respectively. All curves in Figure 4 are normalized in relation to the value of the radial component of magnetization observed on ideal PMs, *i.e.*, by employing *B_rem_*/*μ_o_* as the basis of the normalization.

Figure 4 indicates that the mean value of the circumferential component of the magnetization is zero. Actually, it is so regardless of the number of segments. It can also be understood that the amplitude of the circumferential component will be greater the smaller the number of segments with parallel magnetization.

On the other hand, the radial component has its mean value reduced owing to the employment of segments with parallel magnetization. For four magnets the mean value of the radial component of normalized magnetization is 0.901 times the peak value, while for eight segments the mean value is 0.974 times the peak value. Thus, the greater the number of segments, the greater the mean value of radial component will be; therefore, closer to ideal.

**Figure 5.**
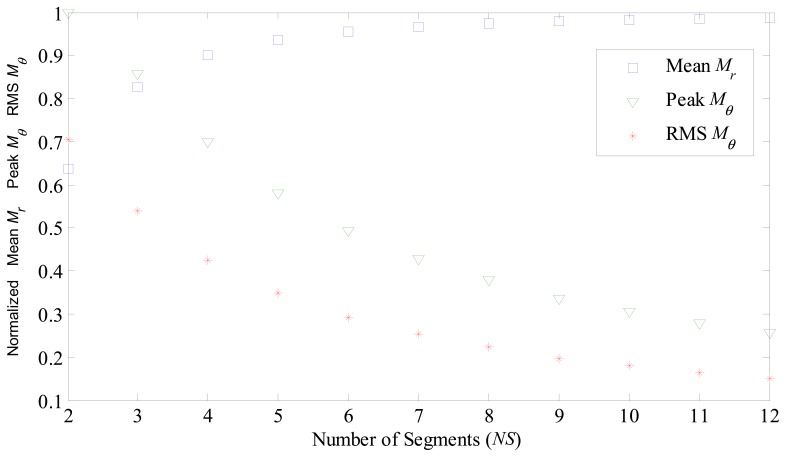
Normalized mean radial component of magnetization for parallel magnetized ring-shaped magnets, and normalized peak value and RMS value of circumferential component for 2 to 12 segments of permanent magnets.

Figure 5 shows the calculated mean value of the radial component of magnetization, peak and RMS value of circumferential component of magnetization, *versus* the number of segments forming a ring. Once again, all values in Figure 5 were normalized in relation to the value of ideal radial magnetization.

## 4. Finite Element Analysis

A finite element model of the actuator shown in Figure 2, with the parameters listed in Table 1, was analyzed using ANSYS Maxwell finite element package. The characteristics of the PMs considered by the simulation are described in Table 2, once they are the same as the ones of the prototype.

### 4.1. 3D Finite Element Model

**Figure 6.**
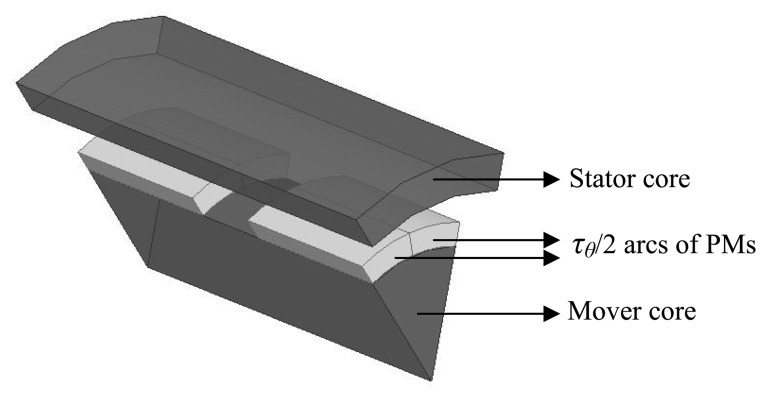
3D model implemented with a finite element analysis package using *τ_θ_* symmetry.

Giving the distribution of the magnetic flux vector, it was necessary to build a 3D finite element model. That can require a high number of elements in order to obtain a smooth distribution of flux density vectors. As a result, it can be time consuming to run such a model and that requires appropriate computing means. In this case, it was feasible to use the symmetry of the actuator to simplify its finite element model since the magnetic distribution is equal in each symmetrical region. An illustration of the adopted axisymmetric model is shown in Figure 6, where coils were suppressed in order to show more clearly the permanent magnets segments. It is a representation of a sector *τ_θ_* passing in between the middle of two adjacent permanent magnets. This approach allows one to increase discretization and therefore to obtain more accurate results without the need to represent the entire actuator by its finite element model.

The FEA package creates tetrahedral elements and allows setting convergence as a function of energy error and restriction on mesh length. In order to obtain an accurate result the maximum internal energy error was set to 0.1%. Also, a refinement on the mesh was applied to restrict the maximum length of the elements to 3 mm in the air gap, magnets and coils. As a result, the total number of elements per region is giving in Table 3 for the initial mesh and for the convergence obtained with two adaptive passes.

Before evaluating the flux density distribution, it is possible to observe that, in a slotless cylindrical actuator, only the radial component of the vector magnetic flux density ***B*** in the air gap produces axial force as giving by:

(6) $$F=\int_{V}J\times Bdv=\int_{V}JB_{r}\left(r,\theta ,z\right)dv=J\int_{V}B_{r}\left(r,\theta ,z\right)dv$$

where ***J*** is the electric current density vector, *J* is the module of the ***J*** vector, *V* is the volume of the coil, and *B_r_* is the spatial distribution of the magnetic flux density component in the radial direction.

**Figure 7.**
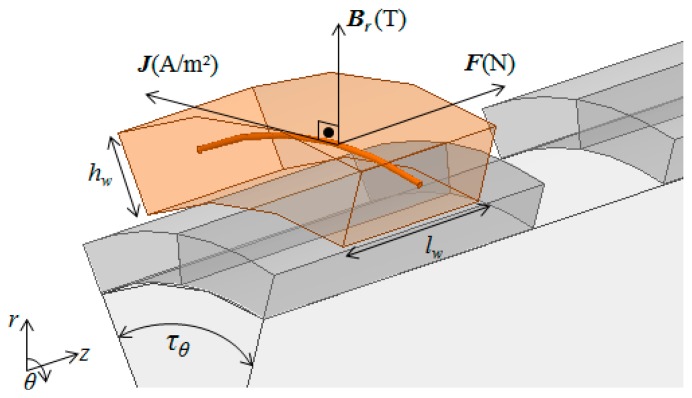
Vectors of radial component of magnetic flux density *B_r_*, electric current density *J* and force *F* over a generic electric conductor.

As illustrated by Figure 7, ***J*** in a generic conductor of the coil is always orthogonal to the radial component of the vector ***B***; therefore, the cross product ***J*** × ***B*** can be substituted by a simple multiplication *JB_r_*, according to Equation (6). The resultant force produced by each coil is giving by the *JB_r_* product integrated over the volume of the coil. The total axial force produced by the actuator is giving by the force computed by the axisymmetric model multiplied by the number of segments.

### 4.2. Air Gap Magnetic Flux Density

**Figure 8.**
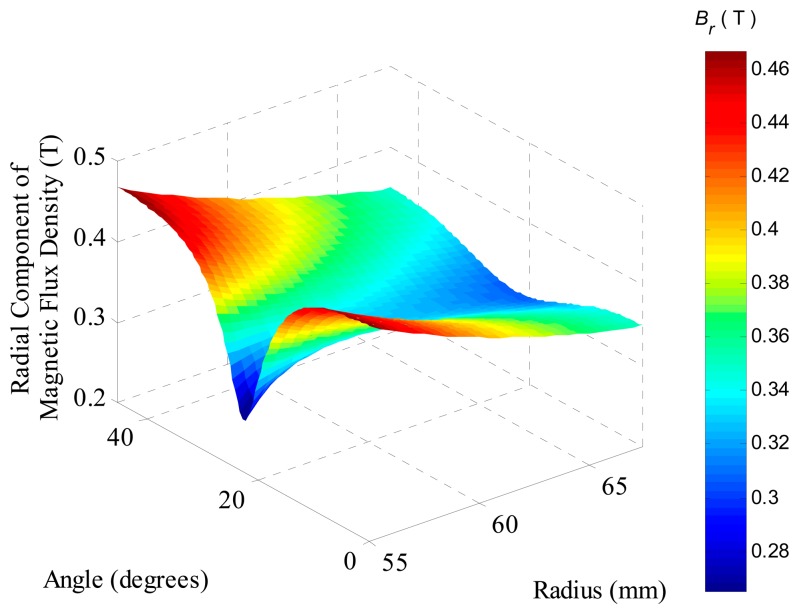
Radial component of magnetic flux density in the region of the coils at an axial section located at *τ_m_*/2 of the actuator with eight segments of parallel magnetization.

The resultant radial component of the magnetic flux density evaluated by FEA over an axial section located in the middle of permanent magnets, *i.e.*, at *τ_m_*/2, of the model, which employs the same parameters as the prototype, is shown in Figure 8. It can be observed that the component presents a sort of valley centered at an angle of 22.5°, which represents the region where two adjacent segments of permanent magnets touch each other. Furthermore, as expected, the radial component decreases as the radius is increased due to the specific topology with internal PMs, where the air gap has a crescent area as the radius increases. The mean value of the radial component of the surface shown in Figure 8 is 0.370 T.

**Figure 9.**
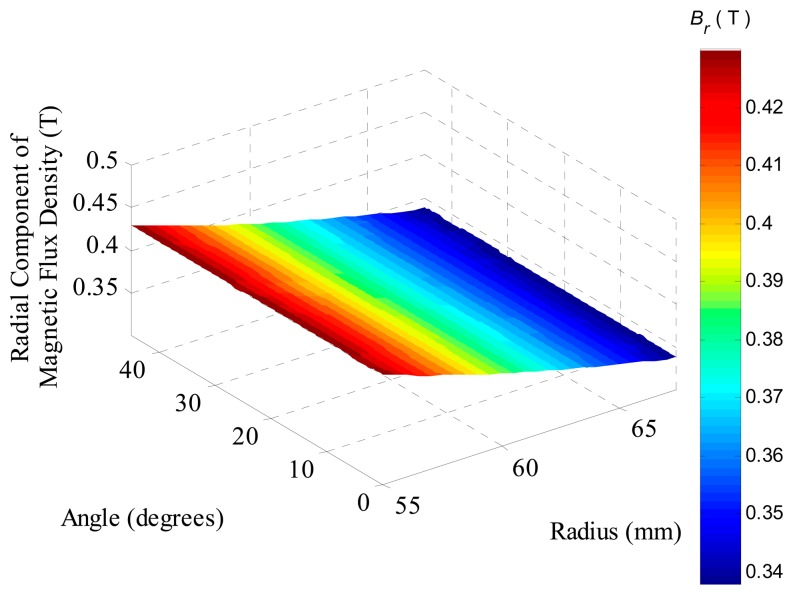
Radial component of magnetic flux density in the region of the coils at a section located at *τ_m_*/2 of the actuator with ideal radial magnetization.

A graph equivalent to the one presented in Figure 8, but for permanent magnets with ideal radial magnetization, is shown in Figure 9. It is possible to observe that the radial component of flux density is constant through the angle range, decreasing only with the increase of the radius. This distribution is expected when an ideal radial magnetization is applied to the permanent magnets for simulation. The mean value of the radial component in the ideal case is 0.378 T. The ratio between the mean value of *B_r_* for the actuator with eight segments with parallel magnetization, and the mean value of *B_r_* for the actuator with ideal radial magnetization is 0.979.

**Figure 10.**
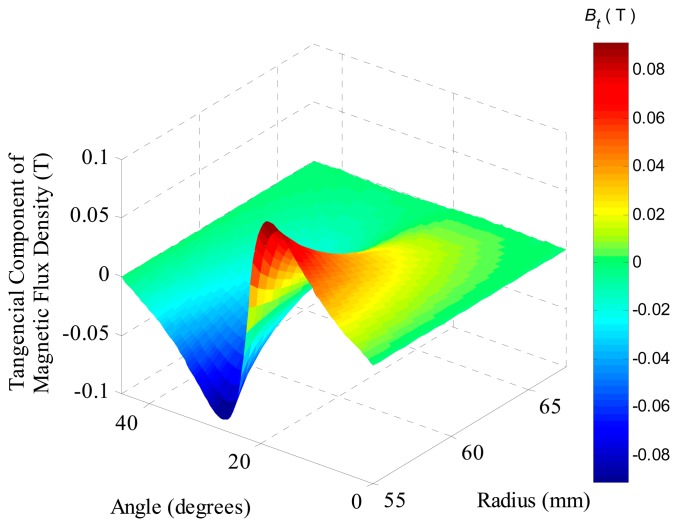
Tangential component of magnetic flux density in the region of the coils at an axial section located at *τ_m_*/2 of the actuator with eight segments of parallel magnetization.

Figure 10 also shows that the component is higher close to the permanent magnets and tends to zero near the stator core. This effect is due to the fact that the flux lines follow the path of least magnetic reactance and for this reason they align in the radial direction in the air gap. In this figure it can be observed that the mean value of the tangential component is zero.

**Figure 11.**
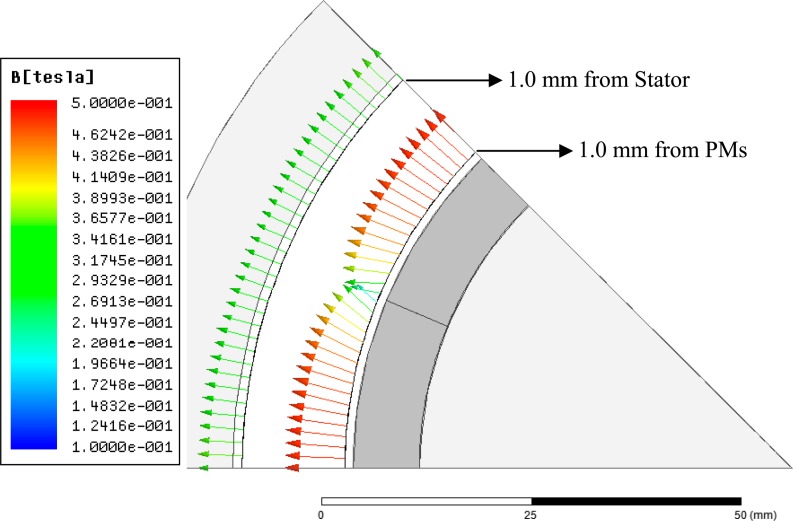
Axial view at *τ_m_*/2 of the vector *B* in the air gap at 1.0 mm from the permanent magnets and at 1.0 mm from the stator core.

The analysis of the Figures 8 and 10 is more easily understood if one looks at the ***B*** vector drawn over two lines in the air gap: one line at 1 mm from the PMs and another line at 1 mm from the stator core, as illustrated by Figure 11. Close to the stator core, vector ***B*** has only radial components, while close to the permanent magnets, especially where two adjacent magnets touch each other, the vector ***B*** has components in the tangential direction.

The axial components of the vector ***B*** do not contribute to produce force in the direction of movement either, although it is known that, in the air gap, the vector ***B*** may also have components in that direction. These components exist, however, for either radial or parallel magnetization, and for this reason it will not be studied here, since it is not a part of the scope of this paper.

### 4.3. Axial Force as a Function of the Number of Segments

**Figure 12.**
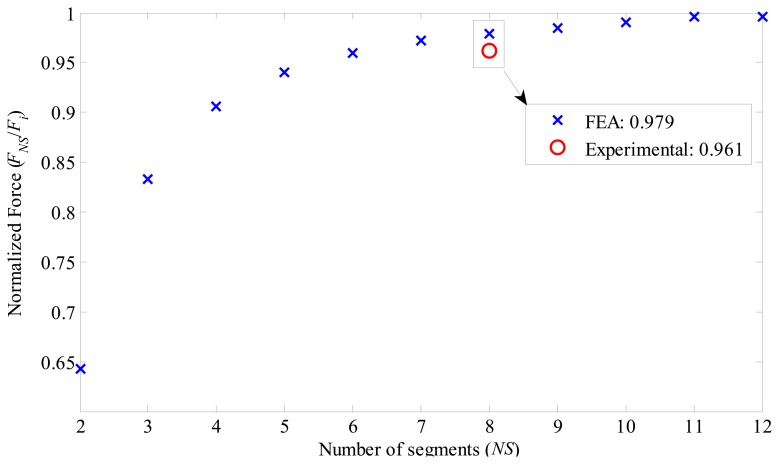
Results of normalized axial force produced by the actuator with parallel magnetized permanent magnets. Values were normalized in relation to an actuator with ideal radial magnetized permanent magnets.

Another important analysis is the total axial force produced by the actuator when the number of segments is varied. The simulation results are shown in Figure 12. In this graph, the force is normalized in relation to the force produced by an actuator with ideal radial magnetization, *i.e.*:

(7) $$\tilde{F_{NS}}=\frac{F_{NS}}{F_{i}}$$

where *F_NS_* is the normalized force produce by an actuator equipped with *NS* segments, *F_NS_* is the force produce by an actuator equipped with *NS* segments, and *F_i_* is the force produce by an actuator equipped with ideal radial magnetization.

For the analysis, all variables were kept constant, except the number of segments. These were varied from two up to twelve segments. In Table 2, it is informed that the arc has 45°, which is for the case of eight segments of the prototype. However, for FEA the arc varies with the number of segments from 30° for twelve segments up to 180° for two segments.

For an applied current density of 1 A/mm^2^ through the conductors of the prototype, the applied current density in FEA must be corrected by a fill factor of 0.586, which was observed experimentally in the coil windings. Therefore, a current density of 0.586 A/mm^2^ was applied to the FEA model, resulting in a total axial force *F_i_* of 106.6 N. The current density of 1 A/mm^2^ was applied because this leads to a low level of armature reaction and avoids saturation of ferromagnetic material. As saturation is not present, any difference in between the actual magnetization curve of the material employed within the prototype and the magnetization curve considered by the FEA model is less significant; therefore, makes it more appropriate for the study of magnetization effects.

Figure 12 indicates the reduction of the produced force in relation to the number of segments. For example, an actuator equipped with two segments produces 64% of the force that an actuator equipped with PMs with ideal magnetization produces, *i.e.*, *F̃*_2_ is 0.64, which represents a reduction of 36% in relation to the ideal. This reduction drops to approximately 1% for an actuator with twelve segments. Figure 12 also shows the experimental result on the graph and compares it to the results obtained with simulation. While simulation estimated *F̃*_8_ equals to 0.979, *F̃*_8_ equals to 0.961 was obtained experimentally. This relative small difference is discussed in the next section.

### 4.4. Parametric Analysis

In order to obtain a general overview of the effect the magnetization pattern has on the performance of a linear slotless cylindrical actuator, so that the work is not limited to the specific geometrical design under study, two parametric analyses were carried out. The first one varies the ratio between permanent magnet length in the axial direction *τ_m_*, and the pole pitch in the axial direction *τ*. The second analysis varies the ratio between the magnetic gap radial length (*R_s_* – *R_m_*) and the permanent magnet radial length (*R_m_* – *R_c_*). For both simulations, a constant magnetic permeability was set for the stator and mover cores so that saturation effects were not present; therefore, they do not affect the specific analysis. The simulations were also carried out for an actuator equipped with permanent magnets with ideal radial magnetization and for another one equipped with eight segments of parallel-magnetized permanent magnets.

#### 4.4.1. Normalized Axial Pole Pitch

**Figure 13.**
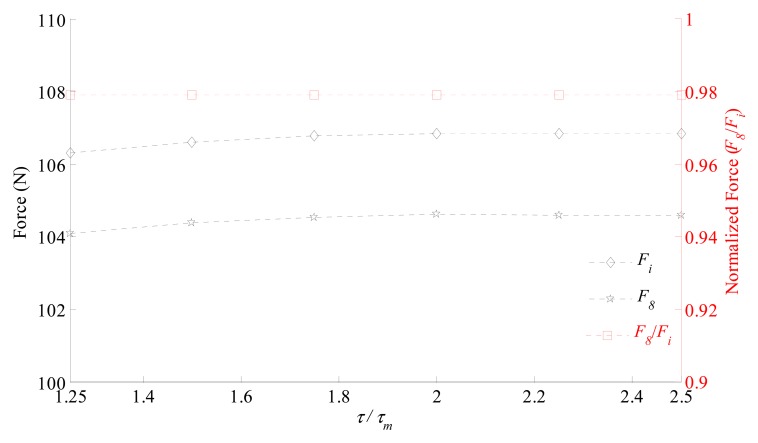
Normalized force (*F̃*_8_ = *F_8_*/*F_i_*) and axial force for an actuator with ideal magnetization (*F_i_*) and for an actuator with eight segments with parallel magnetization (*F_8_*) as function of the ratio between permanent magnet length in the axial direction *τ_m_* and the pole pitch in the axial direction *τ*.

In order to evaluate the effect of the axial pole pitch, the ratio *τ/τ_m_* was varied from 1.25 up to 2.5. That was realized by varying *τ* and keeping *τ_m_* constant. By this way, the volume of the permanent magnets was kept constant and both the stator and mover cores axial lengths varied proportionally. An electric current density of 1 A/mm^2^ was applied. The results of the static axial force *F_i_*, *F*_8_, as well as the normalized force *F̃*_8_ are shown in Figure 13.

Although there is a variation in the resultant force with the variation of *τ/τ_m_*, the normalized force, *F̃*_8_, is nearly constant, which also suggests that the variation is not affected by the magnetization pattern. In a similar way as the analysis of the armature reaction, the ratio *τ/τ_m_* has influence in the flux density distribution on the axial direction, so that in the *rθ* plane it is affected equivalently for both magnetization patterns.

#### 4.4.2. Normalized Magnetic Gap

**Figure 14.**
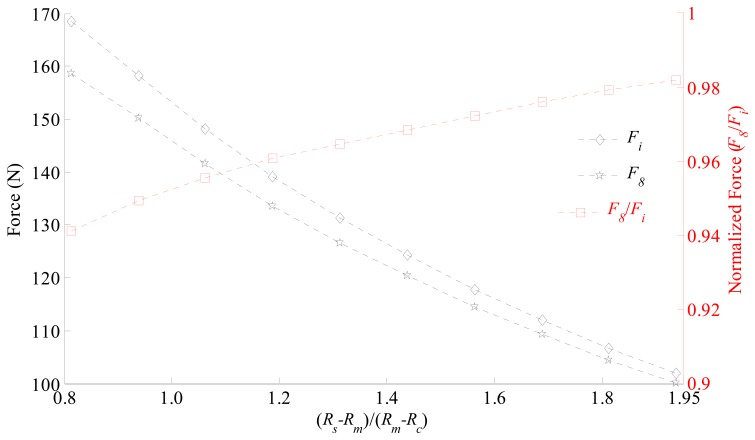
Normalized force (*F̃*_8_ = *F_8_*/*F_i_*) and axial force for an actuator with ideal magnetization (*F_i_*) and an actuator with eight segments with parallel magnetization (*F_8_*) as a function of normalized magnetic gap (*R_s_* − *R_m_*)/(*R_m_* − *R_c_*).

The magnetic gap normalized in relation to the radial length of the permanent magnets (*R_s_* – *R_m_*)/(*R_m_* – *R_c_*) affects the axial force differently for radial and parallel magnetization, once *F̃*_8_ is not constant, as it can be observed in Figure 14. For actuators with smaller magnetic gap there is a significant presence of tangential components of flux density while in actuators with a bigger magnetic gap, even though the tangential component exists, it has a smaller influence on the mean value of *B_r_*. This result is expected because of the distribution of ***B*** on the *rθ* plane, as shown in Figure 11.

The corresponding parametric simulation was carried out maintaining a constant magnetomotive force (MMF) applied to the coils while the geometrical parameters as given in Table 1 were kept constant, except for *R_s_*. For the sake of reference, the normalized magnetic gap of the prototype is 1.8125. As *R_s_* increases, the outer radius of the coils also increases, leading to a larger conduction area. On the other hand, as *R_s_* increases the flux density in the magnetic gap decreases, leading to lower levels of force. The MMF applied to each coil to perform this parametric simulation was 375 A, which corresponds to a current density of 1 A/mm^2^ for the prototype's topology. It should be noted that as *R_s_* decreases the current density increases and *vice versa*.

## 5. Experimental Results

In order to evaluate the effects of parallel magnetization on the prototype with eight segments, measurements of static axial force, normal component of magnetic flux density, and tangential component of magnetic flux density in the air gap at were carried out. In this section, the experimental results are compared with the FEA results.

**Figure 15.**
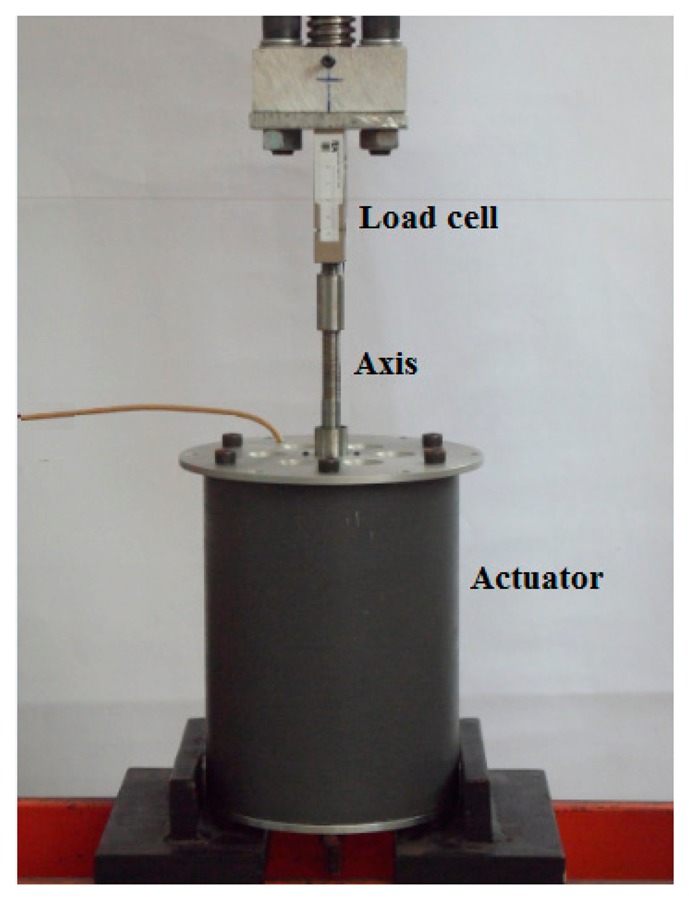
Prototype placed on the test bench for the measurement of static axial force by a load cell.

**Figure 16.**
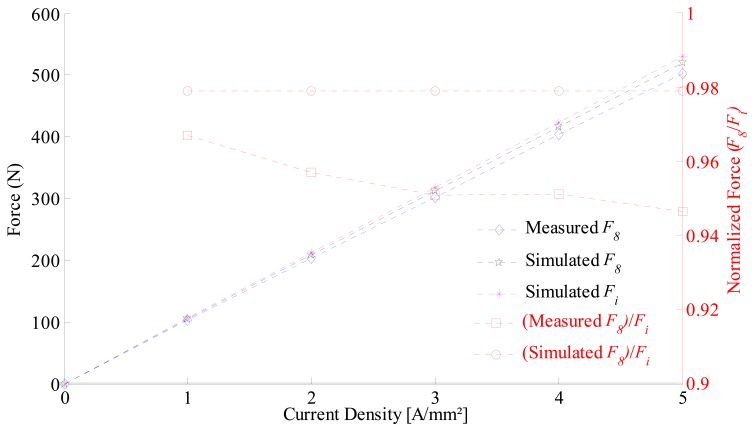
Force *versus* current density: measured on the prototype (Measured *F_8_*) and simulated for an actuator with ideal radial magnetization (Simulated *F_i_*) and for an actuator with eight segments with parallel magnetization (Simulated *F_8_*). Normalized force ((Measured *F_8_*)/*F_i_*) and ((Simulated *F_8_*)/*F_i_*) *versus* current density.

The static axial force was measured on a test bench with the aid of a load cell as shown in Figure 15. The results of the force are expressed in terms of the arithmetic mean value of five measurements. Figure 16 shows the axial force as a function of the current density applied to the prototype and simulated results for: an actuator with ideal radial magnetization, and an actuator containing eight segments with parallel magnetization. The analysis of the graph allows one to figure out that the force has approximately a linear behavior with respect to the current density, as expected, for both simulated and measured results. The force for the ideal magnetization presents the major inclination, describing a better force-to-current density ratio in relation to the segmented one. The measurements presented a slightly lower force-to-current density ratio when compared with the simulation results. This difference can be explained by considering uncertainties of measurements on current density and force. Additionally, differences between the actual magnetic properties of the cores and of the permanent magnets employed within the prototype and the properties considered by the FEA model lead to minor differences between the curves.

Also, for comparison, in Figure 16 the normalized axial force generated by the prototype and the normalized axial force results obtained by FEA model are presented. Analysis of the curve ((Simulated *F_8_*)/*F_i_*) confirms the non-dependence of the current density in simulation results, once the curve is approximately constant. The curve ((Measured *F_8_*)/*F_i_*) has a non-constant value *versus* current density, presenting a decreasing trend as the current density increases, which can be explained by the nonlinear behavior of (Measured *F_8_*) and by the uncertainties of the instrumentation used and of the magnetic properties of materials.

**Figure 17.**
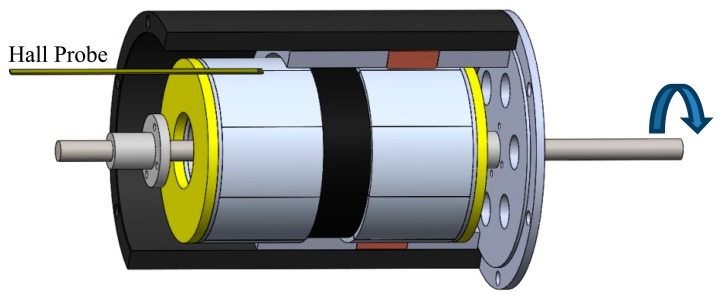
Illustration of the methodology of measurement of radial component of magnetic flux density using a Transverse Hall Probe positioned in the air gap of the actuator while the axis is rotated.

The measurements of the components of ***B*** were performed using a transverse Hall probe as illustrated by Figure 17. The normal and tangential components of ***B*** were measured with the probe positioned at 1 mm and 2 mm from permanent magnets pole surfaces, respectively. These distances were selected once they are the closest position possible from the permanent magnets pole surface in the air gap in radial direction, considering the rectangular shape and dimensions of the transverse probe. The probe was maintained at a static position while the mover, containing the permanent magnets, was rotated. Rotation steps of 1.2 degrees were taken, and the measurements were made three times at each position, so that the result presented is a mean value. A variation of less than 3% at each position was found.

The measurement of the normal component of flux density was carried out by placing the transverse probe orthogonally to the radius of the permanent magnets, while the tangential component of flux density was measurement by placing the transverse probe parallel to the radius of the permanent magnets. In the same way as FEA, the measurements need to be performed only for *τ_θ_*, *i.e.*, a 45° arc for eight segments, because the behavior of the distribution of magnetic flux density in the air gap presents symmetry for each *τ_θ_*.

**Figure 18.**
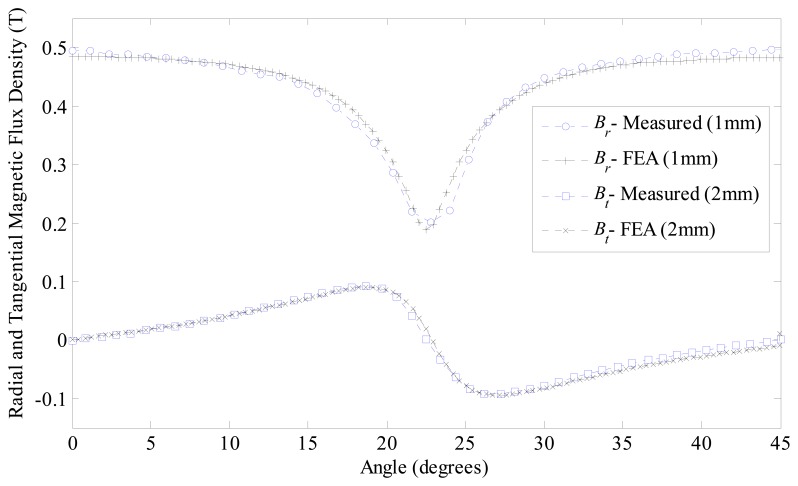
Results of FEA and measurements of normal *B_r_* and tangential *B_t_* components of magnetic flux density as measured in the air gap of the prototype.

Results of normal and tangential components of flux density measured with a model FH54—Magnet Physik^®^ Teslameter are shown in Figure 18. The normal component presents a peak value of 0.49 T and the minimum value is 0.2 T, while the mean value is 0.43 T, which represents a reduction of 12.2% considering that the actuator with ideally radial magnetized permanent magnets would present a constant value equals to 0.49 T. It can be observed that measurements and FEA results show good agreement. The tangential component presents a peak value of 0.09 T and a minimum value of −0.09 T, while the mean value is zero. The peak value of the tangential component tends to decrease as the radial distance from the permanent magnets pole surface in the air gap is increased, while the mean value is always zero.

## 6. Conclusions

Results have shown that segmented permanent magnets produce circumferential components with zero mean value and with amplitude inversely proportional to the number of segments, while the radial component presents mean value proportional to the number of segments. Varying the number of segments from two up to twelve resulted in a variation in the produced force of approximately −36% to −1% respectively, when compared with an actuator with ideal radial magnetized permanent magnets. The prototype with eight segments had its force reduced by 3.9% when compared with simulation results of another one with ideal radial magnetized permanent magnets.

The analysis showed that the segmentation and parallel magnetization of NdFeB permanent magnets employed in cylindrical actuator can be a good alternative, as 3D FEA results showed that for an actuator equipped with eight segments a reduction of only 2.1% on the force is observed in relation to the ideal case. This number drops to less than 1% for twelve segments, being therefore a good alternative to the problem of manufacturing radially magnetized NdFeB ring-shaped magnets.

The analysis also allowed for one to conclude that the ratio between the permanent magnet length in the axial direction and the pole pitch in the axial direction has no effect on the resultant axial force when comparing radial and parallel magnetization. On the other hand, the ratio between the magnetic gap and the permanent magnet radial length has effect on the resultant axial force comparing radial and parallel magnetization, since it affects the radial component of the magnetic flux density in the magnetic gap. The latter ratio should be considered when designing a slotless actuator equipped with parallel-magnetized permanent magnets. It also suggests that devices with small air gaps, such as slotted linear actuators or rotating machines with surface-mounted permanent magnets may have its performance significantly altered by employing permanent magnets with parallel magnetization instead of magnets with ideal radial magnetization. This study, however, may be carried out in a future work.

## Figures and Tables

**Table 1.** Constructive parameters of the actuator.

Property	Unit	Value	Description
*τ_θ_*	rad	*π*/4	PMs segment angle
*τ_m_*	mm	60	PMs length in axial direction
*τ*	mm	90	Pole pitch in axial direction
*R_c_*	mm	45	Stator core outer radius
*R_m_*	mm	53	PMs outer radius
*R_w_*	mm	55	Winding inner radius
*R_s_*	mm	67.5	Mover core inner radius
*l_w_*	mm	30	Axial length of the windings

**Table 2.** Characteristics of the NdFeB permanent magnets.

Material	Arc *τ_θ_*	Inner Radius *R_c_*	Outer Radius *R_m_*	Remanent Flux Density *B_rem_*	Maximum Energy Product	Axial Length *τ_m_*	Density
NdFeB N40	45°	45 mm	53 mm	1.3 T	318.4 kJ/m^3^	60 mm	7.5 g/cm^3^

**Table 3.** Number of elements of 3D FEA Model.

Pass	Coils	Mover Core	Stator Core	Magnets	Air Gap	Total
Initial	51,876	7,306	10,776	54,129	179,062	303,149
Final (2th pass)	55,301	25,499	32,964	76,213	204,125	394,102
